# Oblique effect in visual mismatch negativity

**DOI:** 10.3389/fnhum.2013.00591

**Published:** 2013-09-23

**Authors:** Endre Takács, István Sulykos, István Czigler, Irén Barkaszi, László Balázs

**Affiliations:** ^1^Research Centre for Natural Sciences, Institute of Cognitive Neuroscience and Psychology, Hungarian Academy of SciencesBudapest, Hungary; ^2^Faculty of Education and Psychology, Eötvös Loránd UniversityBudapest, Hungary; ^3^Department of Cognitive Psychology, Institute of Psychology, Eötvös Loránd UniversityBudapest, Hungary

**Keywords:** visual mismatch negativity (vMMN), event-related potential (ERP), unconscious processing, attention, oblique effect, oddball paradigm

## Abstract

We investigated whether visual orientation anisotropies (known as oblique effect) exist in non-attended visual changes using event-related potentials (ERP). We recorded visual mismatch negativity (vMMN) which signals violation of sequential regularities. In the visual periphery unattended, task-irrelevant Gábor patches were displayed in an oddball sequence while subjects performed a tracking task in the central field. A moderate change (50°) in the orientation of stimuli revealed no consistent change-related components. However, we found orientation-related differences around 170 ms in occipito-temporal areas in the amplitude of the ERPs evoked by standard stimuli. In a supplementary experiment we determined the amount of orientation difference that is needed for change detection in an active, attended paradigm. Results exhibited the classical oblique effect; subjects detected 10° deviations from cardinal directions, while threshold from oblique directions was 17°. These results provide evidence that perception of change could be accomplished at significantly smaller thresholds, than what elicits vMMN. In Experiment 2 we increased the orientation change to 90°. Deviant-minus-standard difference was negative in occipito-parietal areas, between 120 and 200 ms after stimulus onset. VMMNs to changes from cardinal angles were larger and more sustained than vMMNs evoked by changes from oblique angles. Changes from cardinal orientations represent a more detectable signal for the automatic change detection system than changes from oblique angles, thus increased vMMN to these “larger” deviances might be considered a variant of the magnitude of deviance effect rarely observed in vMMN studies.

## Introduction

Oblique effect, a well-known phenomenon in visual orientation research, denotes that the nervous system is more sensitive to stimuli of cardinal (vertical and horizontal) than oblique orientations. Various experimental methods demonstrate this anisotropy, e.g., contrast sensitivity for gratings (Campbell et al., 1966; Caelli et al., 1983), visual acuity (Berkley et al., 1975), vernier acuity (Corwin et al., 1977), setting stimuli parallel (Andrews, 1967) and reproduction of stimulus orientation (Gentaz et al., 2001).

The oblique effect most likely originates from the visual cortex (Li et al., 2003, but see Vidyasagar and Urbas, 1982). In a wide range of mammal species more cells respond preferably to cardinal than to oblique stimuli in the visual cortex (Mansfield, 1974; Levitt et al., 1994; Coppola et al., 1998; Li et al., 2003; Xu et al., 2006). The fact that oblique effect emerges if light is projected straight to retina indicates that not the optics of the eyeball or pupil is responsible for the effect (Campbell et al., 1966; Mitchell et al., 1967).

In humans, larger fMRI response was registered to cardinal than to oblique stimuli in V1 (Furmanski and Engel, 2000). Using event-related potentials (ERP) unequal responses have been obtained to cardinal and oblique orientations in steady state potentials (Maffei and Campbell, 1970; May et al., 1979; Skrandies, 1984; Moskowitz and Sokol, 1985); transient ERPs (Yoshida et al., 1975; Arakawa et al., 2000; Proverbio et al., 2002), and MEG (Koelewijn et al., 2011).

Orientation anisotropies were also demonstrated in visual search. In these experiments an oblique stimulus pops out more easily among vertical stimuli, than a vertical stimulus among oblique stimuli (Treisman and Gormican, 1988; Cavanagh et al., 1990). According to the interpretation by Treisman and Gormican, 1988, the visual system treats vertical lines as default, primary value, while oblique lines carry an additional feature (vertical plus a deviancy from vertical). These features are perceived preattentively, without the need of individual examination of every element in the display. On the contrary, the lack of features could only be detected with serial inspection of every stimuli, so increasing the number of distractor elements monotonically increases the reaction time. These results imply that there are essential differences between oblique and vertical orientations. It is important to note that the direction of the asymmetry switches if an aperture is placed over the display having the same orientation as the oblique stimuli, i.e., in this case the vertical stimulus pops-out. However, installing a rounded aperture which is neutral in orientation, oblique stimulus pops-out again, demonstrating that the basis of the phenomenon is the oblique effect, but environmental clues have also important roles. Others have pointed out the influence of vestibular and somatosensory input (Marendaz, 1998; Lipshits and McIntyre, 1999).

In the majority of papers dealing with the oblique effect, stimuli were in the focus of attention, however, the visual search anisotropy indicates that the oblique effect may also be present at the pre-attentive levels. Investigation of the automatic visual change-detection may also underpin that oblique effect is a fundamental phenomenon in visual perception.

Automatic, unconscious deviance-detection is indicated by the auditory (MMN, for review see Näätänen and Winkler, 1999; Näätänen et al., 2007) and visual mismatch negativities (vMMN, for review see Pazo-Alvarez et al., 2003; Czigler, 2007; Kimura et al., 2011). VMMN is usually investigated in the passive oddball paradigm, where standard stimuli are infrequently replaced by deviant stimuli. VMMN might be recorded in various experimental conditions. In one subset of experiments, vMMN-related stimuli are presented in the unattended, task-irrelevant part of the visual field, while subjects are engaged in a task presented in the center of the visual field (e.g., Tales et al., 1999; Czigler et al., 2002). In other type of experiments a single object is presented and certain features, like the shape of a line segment's end, are used for the task while some other features, like the orientation of the line, are used for vMMN elicitation (e.g., Kimura et al., 2010a). VMMN also emerges in conditions when subjects perform a primary auditory task concurrently with unattended visual stimuli (e.g., Astikainen et al., 2004). In most cases vMMN is a negative component within the 120–400 ms latency range over posterior areas, identified in the deviant minus standard difference wave of ERPs. Auditory and visual MMN is considered to emerge whenever the regularity of the incoming discrete elements is automatically registered, and as a result of comparison processes the violation of the regularity by a new event is detected (Winkler and Czigler, 2012). Upon detecting such mismatch, MMN or vMMN emerges reflecting a prediction error.

At least one portion of both the auditory and visual MMN originates from sensory areas of the brain. Studies aimed to localize vMMN (Yucel et al., 2007; Kimura et al., 2009; Urakawa et al., 2010; Müller et al., 2012) are in agreement that it has generators in the visual cortex. Deviant-related negative components on occasion found in anterior electrode sites (Czigler et al., 2004; Clery et al., 2012), frontal sources have been demonstrated as well with fMRI (Clery et al., 2013; Cléry et al., 2013) and LORETA (Kimura et al., 2009). VMMN could be elicited with simple visual deviances, such as motion direction (Pazo-Alvarez et al., 2004), orientation, spatial frequency (Heslenfeld, 2003), color (Czigler et al., 2002), or shape (Maekawa et al., 2005). Studies utilizing orientation change are relatively numerous (Astikainen et al., 2004, 2008; Czigler and Pató, 2009; Flynn et al., 2009; Kimura et al., 2009, 2010a; Czigler and Sulykos, 2010; Sulykos and Czigler, 2011; Sulykos et al., 2013).

In this study we set out to investigate the possibility of orientation anisotropies in vMMN. In a series of experiments we examined whether the system underlying vMMN was more sensitive to orientation deviations from cardinal than from oblique angles. In the first experiment we used a modest change of orientation (50°). While subjects performed a visuomotor tracking task in the center of the visual field, Gábor patches with various orientations were presented in the periphery in an oddball sequence. Infrequent changes in orientation occurred in oblique vs. cardinal as well as in oblique vs. oblique relation. Our main hypothesis was that visual deviance detection is easier if change occurs compared to cardinal than compared to oblique angles, and this will manifest itself in increased vMMN to such changes. This would be in concordance with the findings and theory of Treisman and Gormican (1988). We also expected reduced vMMN to changes from oblique to oblique orientations compared to the other two relations involving cardinal stimuli, as it is suggested by the oblique literature. We also investigated if the oblique effect is manifested in the exogenous ERP components.

We considered the tracking task to be especially appropriate, because this task guarantees continuous and constant attentional demand, while the vMMN-related stimuli are presented as separate, individual objects in a separate part of the visual field. Taking into account the frame effects reported in visual search studies (Treisman and Gormican, 1988), sources of visual orientation were eliminated from the experimental environment by placing a black circular aperture over the computer screen, and by providing no background light in the room.

Following the first electrophysiological experiment we conducted a psychophysical measurement in order to assess the threshold for orientation change detection in an active paradigm with stimuli similar to those used in the passive oddball experiment (i.e., Gábor patches). In addition, the psychophysical measurement allowed us to assess an observation reported earlier, that in contrast to auditory modality where MMN is thought to be elicited by any discriminable difference (Sams et al., 1985; Näätänen et al., 2007), in the visual modality significantly larger differences are necessary to evoke vMMN. For example, in a study by Czigler et al. (2002) pink-black grating changing to red-black grating elicited no vMMN, although in an active paradigm it is easy to detect such color change.

## Experiment 1

### Methods

#### Participants

Seventeen healthy students volunteered in this experiment (12 females, mean age: 22.5 years, age range: 18–32 years) either for modest financial compensation or for course credit. All subjects had normal or corrected-to-normal vision and have given written informed consent after the nature of the experiment had been explained to them. The experiment was approved by the Joint Ethical Committee of the Hungarian Psychology Institutes.

#### Stimuli and procedure

**Figure 1:**
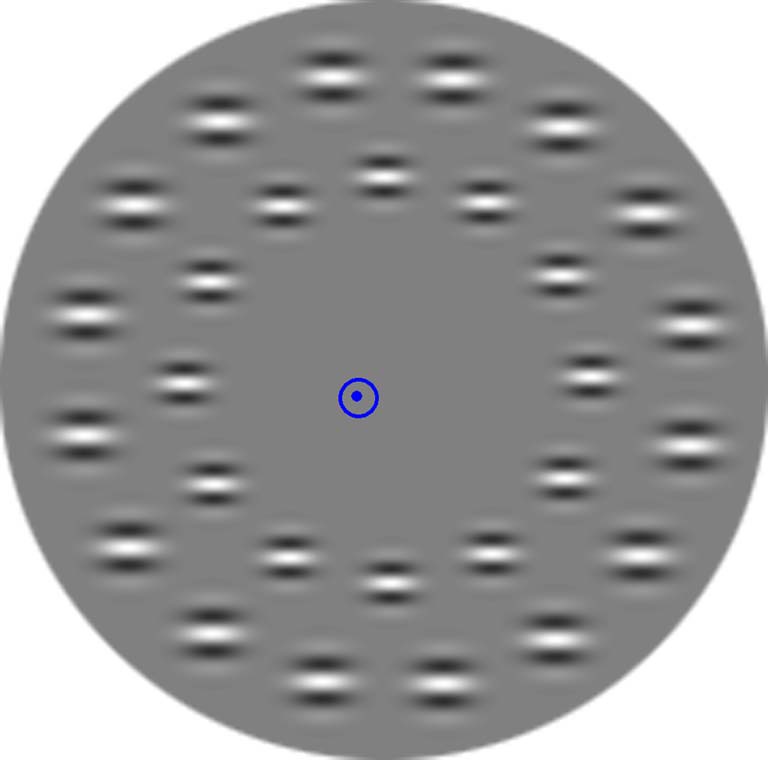
**An example of the display**.

***Task-irrelevant stimuli.*** Task-irrelevant stimuli were Gábor patches (circular grayscale images of Gaussian-windowed sinusoidal gratings; Gaussian standard deviation: 0.17; phase: 45°; trim-value: 0.25, spatial frequency: 3) in two concentric circles (see Figure 1). A circular aperture (radius: 6.2°) was placed over the monitor in order to remove all external orientation clues. The first circle from the center of the screen had 12 patches (diameter: 1.6°). The second, outer circle consisted of 16 patches (diameter: 1.9°). Measured from the center of the screen to the center of the patches, the distance was 3.4 and 5.2°. Care was taken to avoid that the inner and outer circle's patches create radiant lines that could ground for orientation. The background was gray (3.1 cd/m^2^). Stimulus display time was 100 ms, inter-stimulus time was 450 ± 50 ms random jitter to avoid evoking steady state potentials. ERPs were recorded to these task-irrelevant stimuli.

***Task-relevant stimuli.*** Subjects performed a tracking task in the center in a circular task field (1.3°). They were asked to keep an ever-moving dot inside a small circle by tracking down its moves using a trackball (Kensington, Orbit optical trackball). When the dot was inside the circle, the circle was blue (0.9 cd/m^2^), but in case of getting out, the circle switched to red (6.6 cd/m^2^).

Subjects were seated in a reclining chair in a sound-attenuated room, 1.2 m from an 17′ LCD monitor (refresh rate: 60 Hz). No background light was provided in the room.

Task-irrelevant Gábor patches were placed in a pseudorandom oddball sequence, where standards had 83.1% probability. Deviant stimuli were preceded by 3–7 standard stimuli. In one block there were 374 standard and 76 deviant stimuli. Every block was presented twice. As Table 1 illustrates, eight types of standard-deviant pairs were tested: 0 vs. 50° (left from horizontal) (and vice versa), 22.5 vs. 72.5° (and vice versa), 90 vs. 140° (and vice versa) and 112.5 vs. 162.5° (and vice versa). In total 20 blocks were presented, each were approximately 4 min duration. The order of blocks was counterbalanced across participants.

**Table 1:**
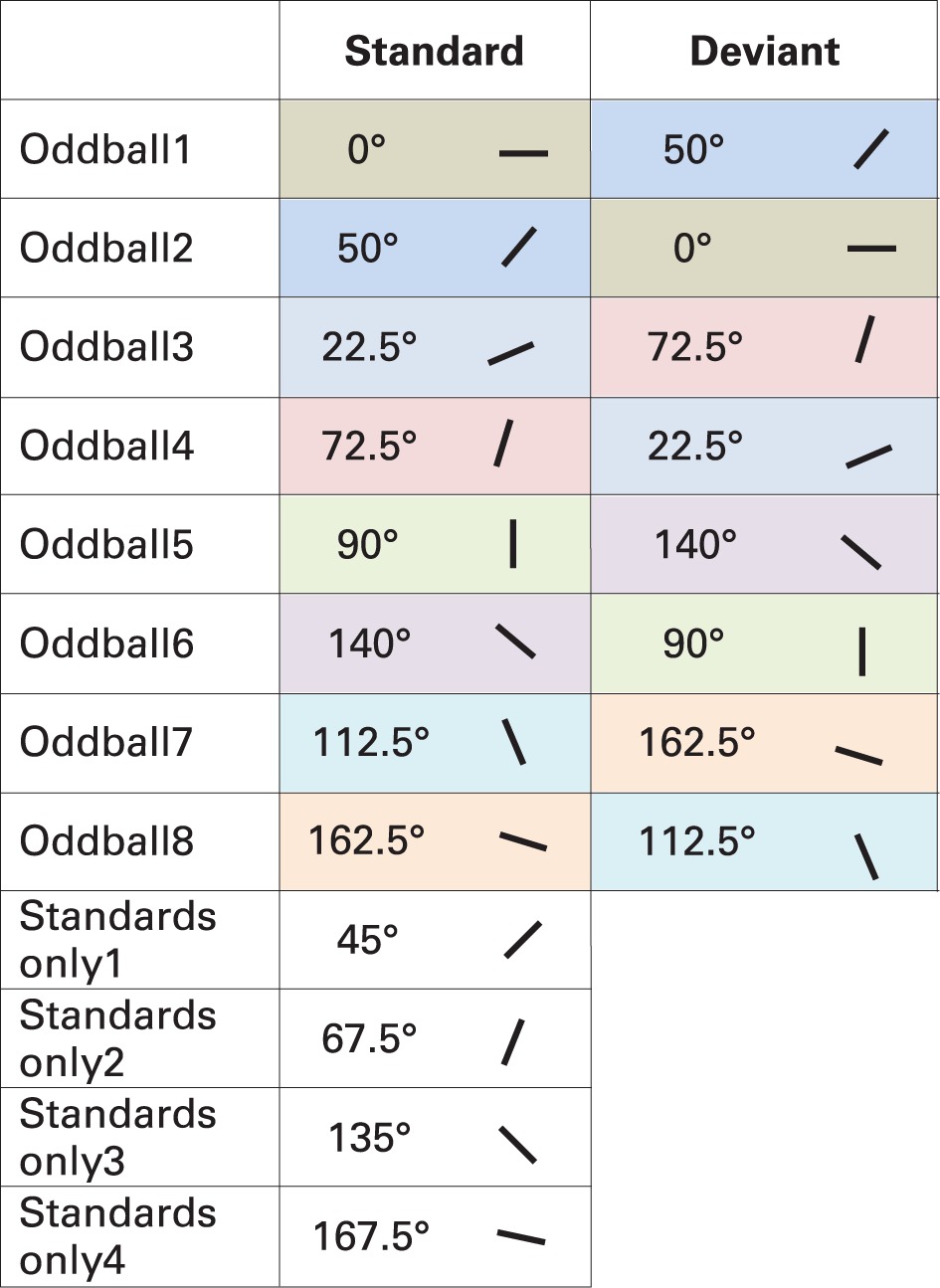
**Stimuli in the oddball and “standards only” sequences in Experiment 1**.

Line segments solely illustrate the orientation of Gábor patches. The standard-deviant pairs highlighted by the same color were used to calculate difference waves.

***ERP recording.*** EEG was recorded with NeuroScan system, DC-100 Hz, sampling rate 500 Hz, with Ag/AgCl electrodes in an elastic electrode cap (EasyCap) on 61 channels from standard locations of extended 10–20 system. Ground electrode was attached to lower forehead, reference electrode was placed on the nose-tip. Reference was offline recalculated to channel average. Horizontal and vertical EOG was recorded with a bipolar montage below and lateral to the eyes. EEG was filtered offline using a bandpass filter of 0.1 and 30 Hz (24 dB/octave slope). EEG and EOG activities were averaged for epochs beginning 100 ms before and extending until 400 ms after stimulus onset. The mean voltage of the first 100 ms served as baseline interval. Epochs containing amplitude changes exceeding 50 μ V at any channel were rejected from analysis. Standards preceded by at least three other standards were averaged. ERPs recorded in “standards only” sequences were all averaged, regardless their positions. After artifact rejection for deviants in average 126.5 epochs (*SD* = 20.0; range: 64–148), for standards 226.7 epochs (*SD* = 36.9; range: 112–267) and for the “standards only” 153.6 epochs (*SD* = 26.8; range: 46–178) were included in the mean for one subject.

***Analysis.*** To analyze change-related activities, we calculated difference waves by subtracting ERPs elicited by the very same stimulus as a deviant and a standard (Kujala et al., 2007). Table 1 depicts how the difference waves were calculated. Pairs of standard and deviant stimuli are highlighted in different colors which were used to calculate the difference waves that formed the basis of further analyses. For instance, difference wave for horizontal stimuli (0°) was calculated by subtracting Oddball1 sequence standard ERPs from Oddball2 sequence deviant ERPs. Though, these ERPs were recorded in separate blocks, this way physically same stimuli were subtracted from each other, the only difference between them was their roles of being a standard or a deviant.

In two conditions stimuli changed from oblique to cardinal^1^ (0 and 90°), in other two conditions from cardinal to oblique (50 and 140°), and in four conditions from oblique to oblique (22.5, 72.5, 112.5, and 162.5°). Additional four orientations (45, 67.5, 135, and 167.5°^2^) were presented in separate blocks without deviants (“standards only” conditions).

Negative going difference-waves were considered to be valid vMMN responses if point-by-point *t*-test (see, e.g., Guthrie and Buchwald, 1991) were significant at 0.05 level at least at two adjacent parieto-occipital channels in five consecutive time points (10 ms) between 100 and 250 ms after stimulus onset.

For orientation-related amplitude differences we compared the mean amplitude of standard stimuli in 40 ms wide time windows centered around the latency of N1b subcomponent on six parieto-occipital channels (PO7, POz, PO8, and O1, Oz, O2), where this component was most evident by visual inspection. For linear regression models we report *R*^2^ coefficient of determination, *F*- and *p*-values.

Tracking task performance was assessed by calculating tracking efficiency, the percent of time when the dot was located inside the circle. Repeated measures ANOVA were performed to compare tracking efficiency in conditions where stimuli changed from cardinal to oblique (50, 140°), from oblique to cardinal (0, 90°) and from oblique to oblique orientations (22.5, 72.5, 112.5, and 162.5°).

Greenhouse-Geisser correction was applied when appropriate. Significant interactions were further specified by Tukey HSD *post-hoc* test. Partial eta squared (η^2^) presents effect size estimates.

### Results

#### Behavioral results

Repeated measures ANOVA on tracking efficiency with factor conditions revealed significant effects, *F*_(2, 34)_ = 3.4, *p* < 0.05, η^2^ = 0.17. Tracking efficiency was 81.4% (*SE* = 1.73%) in blocks where stimuli changed from oblique to cardinal, 80.6% (*SE* = 1.80%) in blocks where stimuli changed from cardinal to oblique, and 81.8% (*SE* = 1.69%) in blocks where stimuli changed from oblique to oblique orientations. *Post-hoc* comparison showed that the latter two conditions differed significantly from each other.

#### ERP results

The response to standard and deviant stimuli displays a positivity-negativity-positivity sequence on Oz channel (see Figure 3) that could be identified as P1-N1a-P2 response. These components peak at 94, 112, and 240 ms, respectively. Between N1a and P2 components at lateral, occipito-temporal channels another negative deflection could be observed with a latency of 170 ms (N1b).

**Figure 2:**
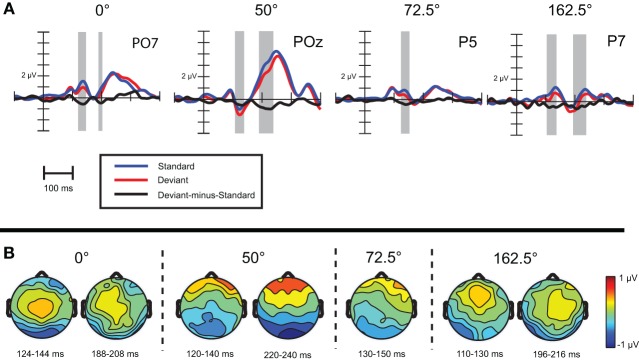
**(A)** Event-related activity and deviant-minus-standard difference potentials at the location having largest amplitude of the difference potentials in Experiment 1. Intervals marked in gray signaled significant deviant-minus-standard differences by point-by-point *t*-tests. **(B)** Topographic voltage maps of the deviant-minus-standard difference potentials.

***Visual mismatch negativity.*** Difference waves for eight stimulus orientations (0, 50, 22.5, 72.5, 90, 140, 112.5, 162.5°) were calculated. Point-by-point *t*-tests revealed only four conditions out of eight, where vMMN emerged. Figure 2 displays grand-average waveforms and topographic voltage maps for vMMN in these conditions. As Table 2 shows, in all four conditions there was an early time interval (latencies between 120 and 140 ms) for vMMN. In three conditions, vMMN appeared also in a later time interval, with peak latency falling between 198 and 230 ms.

**Table 2:** **VMMN in Experiment 1**.

	**Early time interval**			**Later time interval**		
	**Channels**	**Latency (ms)**	**Peak amplitude (μV)**	**Channels**	**Latency (ms)**	**Peak amplitude (μV)**
0°	P5, P7, PO3, PO7, PO4, O1, Oz, O2	134	−0.65	P7, PO7	198	−0.47
50°	P1, P5, Pz, P2, P4 PO3, POz, PO4	130	−0.53	P5, P3, P2, P4, P6, P7, PO3, POz, PO4, P8, PO7, PO8, O1, Oz, O2	230	−1.06
72.5°	P5, PO3, P3	140	−0.45			
162.5°	P5, P7, PO3, PO7, O1	120	−0.53	P5, P7, PO7, O1	206	−0.55

*Channels that exhibited vMMN. Latency and peak amplitude was measured on grand-average waveforms*.

**Figure 3:**
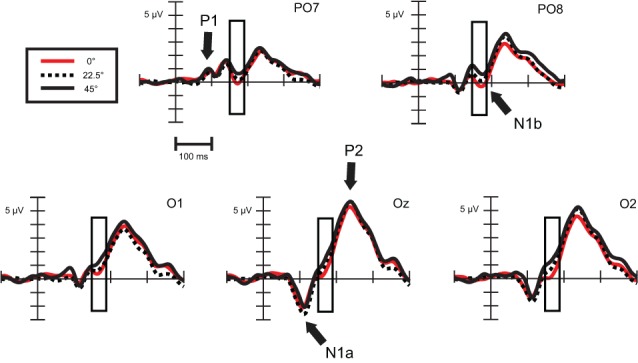
**Exogenous differences at occipital leads in Experiment 1 for standard stimuli**. Significant differences were found in the time range of N1b subcomponent (150–190 ms), which are indicated by the rectangular boxes. P1, N1a, N1b, and P2 components are marked where they are most evident. For the sake of visibility, we display just three angles.

***Exogenous differences.*** We compared ERPs evoked by standard stimuli in the twelve available orientations: 0, 22.5, 45, 50, 67.5, 72.5, 90, 112.5, 135, 140, 162.5, and 167.5°. To simplify the illustration of the orientation effect, on Figure 3 there are only three orientations, a cardinal (0°) and two oblique angles (22.5, 45°).

**Figure 4:**
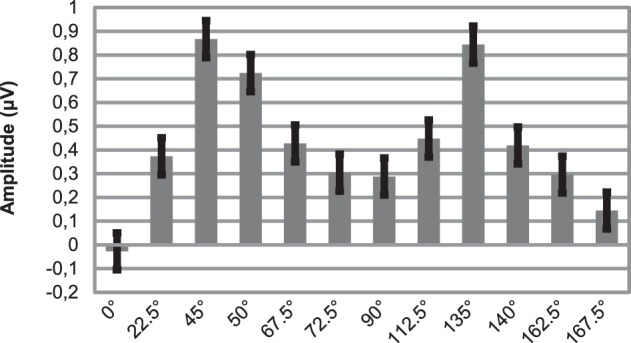
**Mean amplitudes of N1b subcomponent at PO8 location for 12 different orientations**. Note that due to impact of the adjacent P2 wave, responses are positive in voltage. Errors bars represent standard error.

As Figure 3 illustrates, around 170 ms (in the time range of the N1b sub-component at occipital channels) there are orientation-related amplitude differences. Although responses were positive in voltage in most cases, N1b subcomponent is a negativity shaped by the adjacent dominant P2 wave. Figure 4 shows mean amplitudes averaged across subjects at PO8 channel, where N1b component reached its maximum. Amplitudes were highly dependent on the orientation of stimuli.

In order to build a linear regression model, we defined a new variable, deviancy from cardinal orientation, which equals to the difference between the given orientation and closest cardinal orientation (e.g., 72.5° has a 17.5° deviancy from cardinal orientation, because the closest cardinal orientation is 90°). A simple linear regression analysis was conducted at five posterior leads (PO7, O1, Oz, O2, PO8), where we predicted mean amplitudes with the independent variable of deviancy from cardinal orientations. Table 3 displays the results of the regression analyses. The high coefficient of determination (*R*^2^) indicates that the orientation of Gábor patches is a good predictor of the N1b amplitude.

**Table 3:** **Statistics for N1b exogenous differences in Experiment 1**.

	***R*^2^**	***F***	***p***
PO7	0.70	*F*_(1, 10)_ = 23.05	*p* < 0.001
O1	0.61	*F*_(1, 10)_ = 15.33	*p* < 0.01
Oz	0.65	*F*_(1, 10)_ = 18.64	*p* < 0.01
O2	0.73	*F*_(1, 10)_ = 26.89	*p* < 0.001
PO8	0.79	*F*_(1, 10)_ = 38.12	*p* < 0.001

Linear regression analysis.

### Discussion for experiment 1

To conclude, in Experiment 1 vMMNs were evoked sporadically, only in four conditions out of eight. We observed orientation-related amplitude differences in the latency range of occipito-temporal, lateral N1b component, around 170 ms.

Sulykos and Czigler (2011) presented similar Gábor patches in their experiment. Orientation related vMMN was elicited with 130 and 132 ms peak latency at lower and upper visual field stimulation, respectively. The differences found in the earlier time interval (120–140 ms) in the present study correspond to these latency ranges. However, in the present study we obtained vMMN only in half of the conditions, and there was no oblique-related order in the emergence of deviant-related negativity. Since our hypothesis was based on finding valid vMMN responses in all conditions or at least in those involving cardinal stimuli, conclusions pertaining to the existence of oblique effect on vMMN could not be made based on the data of the present experiment.

Contrary to vMMN, amplitude changes of an exogenous component, the N1b suggest the visual system was able to precisely map the orientation of Gábor patches and ERP methods were suitable for detecting these responses. In the light of these results, the lack of reliable vMMN is even more surprising. It is clear that the processing of orientation did not raise difficulties for the visual system, even if stimuli were in the visual periphery and out of the focus of attention.

The small, but significant difference in tracking efficiency between two conditions (changes from cardinal to oblique vs. changes from oblique to oblique) was an unexpected finding. Czigler and Sulykos (2010) demonstrated subtle interactions between the task-relevant and irrelevant stimuli in a similar experimental setup.

In a supplementary experiment we tried to determine the amount of orientation difference that is needed for change detection in an active, attended paradigm. Subjects were required to detect orientation change of Gábor patches while they were reading aloud numbers in the center appearing simultaneously with the patches. Short (100 ms) and simultaneous display of numbers and Gábor patches prevented subjects from using eye movements to fixate on Gábor patches. In this way subjects detected orientation change through peripheral vision, like in Experiment 1. The tracking task used in Experiment 1 would not have provided the required control over subject's eye movements.

Our goal was to reproduce the classical oblique effect with this type of stimulus array, that is Gábor patches with moderately high spatial frequencies in concentric circles. In addition, we could assess the former observation (Czigler et al., 2002) that vMMN could be registered with significantly larger deviances than what could be detected in an active paradigm.

## Psychophysical measurements

### Methods

#### Participants

Eighteen subjects were recruited in this experiment. Five subjects were excluded due to the high number of false alarms that is more than three false alarms in any of the four blocks. An additional subject was excluded due to very low performance in one block. The final cohort therefore consisted of 12 volunteers (7 females, mean age: 21.6 years, age range: 18–30 years). This sample was partly overlapping with the sample of Experiment 1, eight subjects participated in both experiments.

#### Stimuli and procedure

In this experiment the central and peripheral visual field were both task-relevant. The peripheral stimuli were identical to Experiment 1 stimuli, i.e., Gábor patches in two concentric circles were presented. In the center of the display random numbers from 1 to 9 (color: magenta, 7.8 cd/m^2^, size: 0.5°) were presented. The background was gray (3.1 cd/m^2^). The stimulus duration was 100 ms, inter-stimulus interval was 1500 ms.

The peripheral and central stimuli always appeared simultaneously. The task was to read aloud the numbers while detecting the change in the orientation of the Gábor patches. Participants were instructed to press a button with their dominant hand upon detecting any change in the background. Subjects have been video-monitored in real time by a research assistant to make sure they kept reading aloud the numbers.

Gábor patches were arranged in an oddball sequence. Standards were 0° (horizontal), 22.5, 90 and 112.5°. Standard probability was 77.8%. At least 2, at most 5 standards followed a deviant stimulus. Deviant stimuli differed in orientation from standards. Amount of this difference was changing throughout the experiment depending on the subject's response, but deviants were most of the time^3^ oblique orientations. One up, one down staircase sequence was introduced. Until first reversal, step-size was 10°, and then step-size was reduced to 1°. Initial difference was set to 20° counterclockwise from the standard stimuli. Threshold was calculated as the mean of the last six reversals out of total 11 reversals. Subjects were tested in four blocks for each standard orientation.

The order of blocks was counterbalanced across participants. Prior to this experiment, participants performed Experiment 1, then they had a short break while the EEG-cap was removed and they washed and dried their hair.

### Results

**Figure 5:**
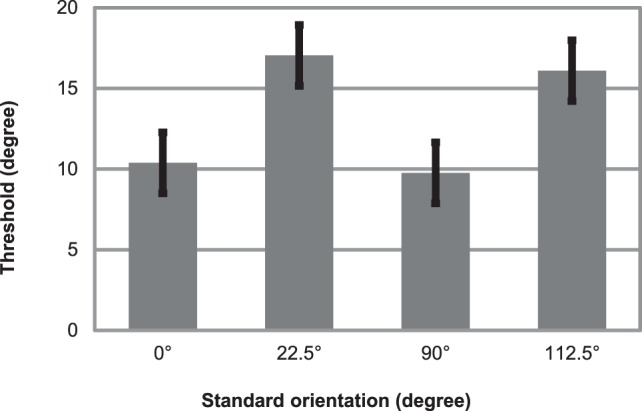
**Mean thresholds for detecting orientation deviants in an oddball sequence of Gábor patches**. Parallel task was to read aloud random numbers appearing in the centrum simultaneously with the Gábor patches. Errors bars represent standard error.

Figure 5 displays mean thresholds. On the mean threshold data of the four conditions we performed a repeated measures ANOVA with factors cardinality (cardinal: 0, 90° vs. oblique 22.5, 112.5°). A cardinality main effect emerged, *F*_(1, 11)_ = 10.6, *p* < 0.01, η^2^ = 0.49, reflecting that thresholds were lower when standards were cardinal orientations (threshold: 10.08°; *SE* = 1.99) compared when standards were obliquely oriented (threshold: 16.57°; *SE* = 2.38).

### Discussion for psychophysical measurements

Thresholds for detecting orientation deviants were 10 and 16.5° in this experiment. Thresholds were significantly lower when standards were cardinal stimuli (compared when standards were oblique orientations), exhibiting the classical oblique effect. This finding is also in line with the results and theory of Treisman and Gormican (1988).

These thresholds, 10 and 16.5° are appreciably smaller than the 50° orientation change that in fact did not elicit reliable vMMN in Experiment 1. These results provide evidence that perception of change in the visual periphery could be accomplished at significantly smaller thresholds, than what elicits vMMN.

One major difference should be noted between this experiment and the vMMN experiment. Although, in this task central vision was occupied with detecting random numbers, subjects still attended consciously to the Gábor patches. The design of our vMMN experiment is intended to prevent subjects from conscious attention towards stimuli used to elicit ERPs. So these two experiments are really different in a major feature (attentive vs. non-attentive processing), that could account for the markedly different results. However, it is possible that the lack of vMMN is attributable to low signal-to-noise ratio that results from presenting too small orientation change (50°) for reliable vMMN emergence. To test this possibility, and as an attempt to record reliable vMMN, in the next experiment we increased the orientation change to 90°.

## Experiment 2

### Methods

#### Participants

Nineteen subjects (11 females, mean age: 21.4 years, age range 19–25 years) participated in this experiment. None of them took part in the previous experiments.

#### Stimuli and procedure

Task-irrelevant stimuli were similar to stimuli in Experiment 1, with the following exceptions. First, Gábor patches were displayed in three circles^4^, the center of the patches in the first circles were 1.9° from the center of display and Gábor-patches were 1.3° in size. The first circle consisted of eight patches. The other two circles and the task-relevant stimuli were identical to Experiment 1.

As shown in Table 4, there were four stimulus conditions: 0 vs. 90° (vice versa) and 45 vs. 135° (vice versa). As every block was repeated twice, there were eight blocks presented altogether, and all of them were intended to measure vMMN.

**Table 4:**
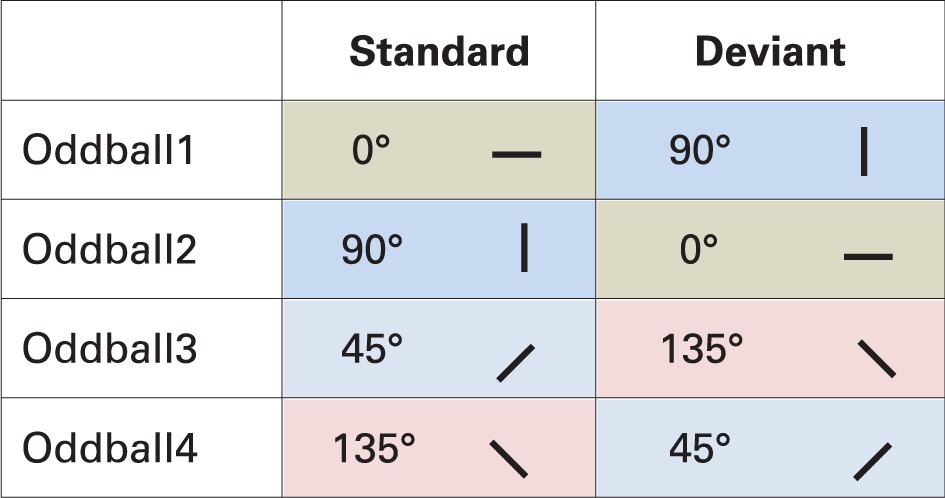
**Stimuli in the oddball sequences in Experiment 2**.

Line segments solely illustrate the orientation of Gábor patches. The standard-deviant pairs highlighted by the same color were used to calculate difference waves.

***Analysis.*** For each subject an average of 134.6 epochs (*SD* = 8.9, range: 99–147) was included in the mean response to deviants, and 249.6 epochs (*SD* = 16.9, range: 173–272) in the standard response. Analysis was identical with Experiment 1, with the following exceptions. For every stimuli condition we determined the latency of the vMMN response, based on the grand average difference waveforms. The latency was measured at the channel where difference wave reached its maxima between 100 and 250 ms. Mean amplitudes of the deviant-minus-standard difference wave were measured around this latency in 60 ms wide windows, in the same time interval for every subjects.

For statistical analyses of vMMN a 2 × 3 grid of parietal and occipital channels were used (PO3, POz, PO4; PO7, Oz, PO8). Repeated measures ANOVA was applied on the mean amplitude values of the difference wave including factors cardinality (cardinal: 0 and 90°; oblique: 45 and 135°), anteriority (anterior: PO3, POz, PO4; posterior: PO7, Oz, PO8) and laterality (left: PO3, PO7; midline: POz, Oz; right: PO4, PO8).

Orientation-related amplitude differences were analyzed in two time-intervals, between 100 and 140 ms for N1a component and between 150 and 190 ms for N1b component.

### Results

#### Behavioral results

Repeated measures ANOVA on tracking efficiency with factor cardinality revealed no significant effects. Tracking efficiency was 78.9% (*SE* = 1.56%) in cardinal blocks and 79.6% (*SE* = 1.59%) in oblique blocks.

#### ERP results

**Figure 6:**
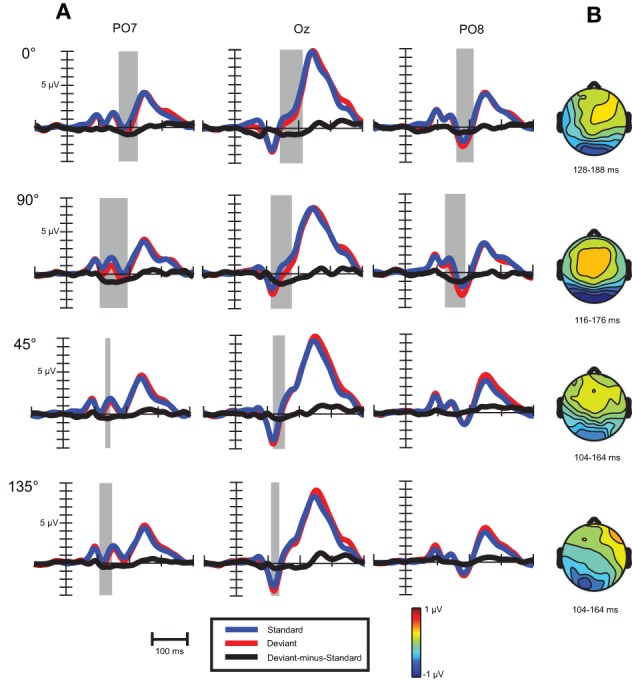
**(A)** Event-related activity and deviant-minus-standard difference potentials at the location having largest amplitude of the difference potentials in Experiment 2. Intervals marked in gray signaled significant deviant-minus-standard differences by point-by-point *t*-tests. **(B)** Topographic voltage maps of the deviant-minus-standard difference potentials in the time-window used for statistical analysis.

As Figure 6A shows, similar waveforms were obtained for standard and deviant stimuli as before. On Oz channel P1-N1a-P2 sequence was elicited, with similar latencies (94, 116, and 240 ms) as in the previous experiment. The occipito-temporal N1b component with 180 ms peak latency was more pronounced in this experiment.

***Visual mismatch negativity.*** In the four conditions (0, 45, 90, and 135°) deviant-minus-standard difference waves were calculated (see Figure 6A). Visual inspection and point-by-point *t*-tests revealed that vMMN responses were present in every condition between 100 and 200 ms, with maxima between 134 to 162 ms (see Table 5). On anterior channels positive components were present with similar latencies as the posterior vMMNs. Around 270 and 340 ms the difference waves were positive with a parieto-occipital maximum scalp-distribution.

**Table 5:** **Mean peak amplitudes and mean latencies used for statistical analyses**.

		**Mean peak amplitude (μV)**	**Mean latency (ms)**	**Mean peak amplitude (μV)**	**Mean latency (ms)**
0°	Cardinal	−0.67	155	−0.50	162
90°				−0.84	148
45°	Oblique	−0.36	135	−0.35	134
135°				−0.37	136

In a repeated measures ANOVA on mean vMMN amplitudes with factors cardinality, anteriority and laterality a main effect of cardinality *F*_(1, 18)_ = 5.3, *p* < 0.05, η^2^ = 0.23 was obtained, revealing more negative amplitudes in response to cardinal stimuli.

For investigating peak latency differences repeated measures ANOVA was performed with the same factors as above. A cardinality main effect emerged, *F*_(1, 18)_ = 55.0, *p* < 0.00001, η^2^ = 0.75, which was due to faster latencies in response to oblique angles. We also found an anteriority main effect, *F*_(1, 18)_ = 17.2, *p* < 0.001, η^2^ = 0.49, reflecting faster latencies at anterior row of channels (142 vs. 148 ms).

**Figure 7:**
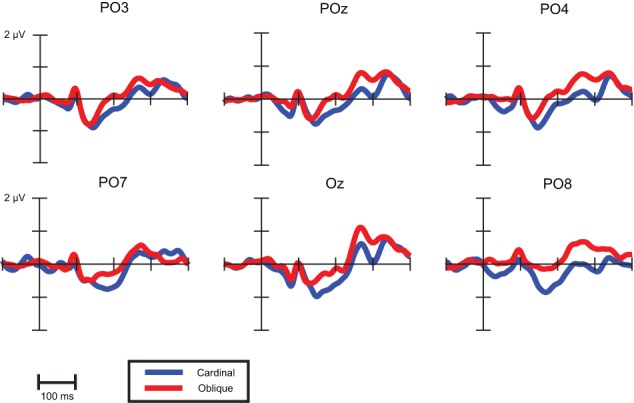
**VMMNs (deviant-minus-standard difference potentials) evoked by cardinal and oblique stimuli**.

The question arises whether latency differences reflect earlier timing of vMMN to oblique conditions. Since both waveforms and topographical voltage maps exhibited close concordance in cardinal (0 and 90°) and oblique (45 and 135°) stimuli conditions, we collapsed these responses, Figure 7 shows these records.

As a descriptive analysis of onset and offset times Table 6 displays the first time points where point-by-point *t*-tests were significant in the time intervals of cardinal and oblique vMMN responses. Differences between these conditions are notable in offset times only, which suggest that latency differences between oblique and cardinal conditions does not imply earlier timing for oblique vMMNs.

**Table 6:** **VMMN onset and offset times (in ms) at parieto-occipital channels based on grand-average waveforms**.

	**PO3**		**POz**		**PO4**		**PO7**		**Oz**		**PO8**	
	**Onset**	**Offset**	**Onset**	**Offset**	**Onset**	**Offset**	**Onset**	**Offset**	**Onset**	**Offset**	**Onset**	**Offset**
**Card**.	112	182	122	168	120	176	106	220	116	196	144	204
**Obl**.	110	160	116	154	116	150	108	156	112	164	NaN	NaN
**Diff**.	2	22	6	14	4	26	−2	64	4	32		

Last row displays difference between these measures (cardinal minus oblique).

**Figure 8:**
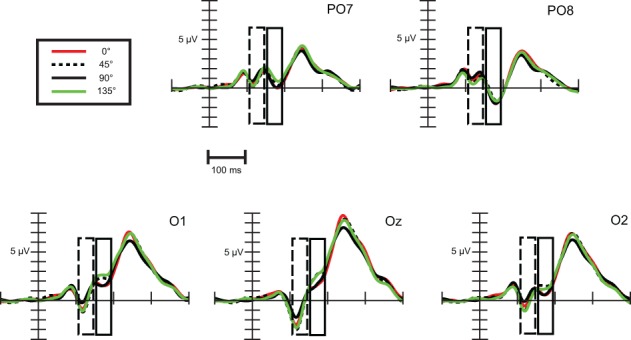
**Exogenous differences at occipital leads in Experiment 2 for standard stimuli**. Significant differences were observed in the time range of N1a (100–140 ms) and N1b subcomponent (150–190 ms), which are indicated by the dashed and solid line rectangular box, respectively.

***Exogenous differences.*** Figure 8 depicts visual evoked potentials to four standards (0, 45, 90, and 135°). Orientation-related amplitude differences were evident already in the time interval of the N1a component around 120 ms post-stimulus. Response amplitudes to vertical (90°) and marginally to horizontal (0°) appeared to be less negative than to oblique orientations.

Repeated measures ANOVA conducted with factors stimulus (0, 45, 90, and 135°) and channels (PO7, O1, Oz, O2, PO8) revealed that differences are present between orientations. Stimulus main effect was significant, *F*_(3, 54)_ = 11.61, ε = 0.89, *p* < 0.0001, η^2^ = 0.39. *Post-hoc* tests inform that it was due to significant differences between amplitudes to 90° and to every other orientation. In addition, a stimulus × channel interaction was found, *F*_(12, 216)_ = 2.64, ε = 0.32, *p* < 0.05, η^2^ = 0.13. According to *post-hoc* comparisons, responses to 90° differed from responses to 0° at O1 and Oz, from responses to 45° at every channel, and from responses to 135° at O1, Oz, O2, PO8 channels. Responses to horizontal (0°) differed from oblique orientations only at Oz (0 vs. 45°) and at PO8 (0 vs. 135°). So we can conclude that around 120 ms N1a amplitudes to vertical orientation were less negative and horizontal orientation exhibited almost negligible difference.

Differences around 170 ms, in the time-interval of the N1b component were much clearer between orientations. We conducted a repeated measures ANOVA with factors stimulus (0, 45, 90, and 135°) and channels (PO7, O1, Oz, O2, PO8). A stimulus main effect emerged, *F*_(3, 54)_ = 8.96, ε = 0.89, *p* < 0.001, η^2^ = 0.33, *post-hoc* tests revealed significant differences between every cardinal-oblique pairs, and no difference between cardinal-cardinal and oblique-oblique pairs. We obtained a stimulus × channel interaction, *F*_(12, 216)_ = 4.36, ε = 0.36, *p* < 0.01, η^2^ = 0.20. *Post-hoc* comparisons between cardinal and oblique orientations showed that differences were significant mainly at the three occipital channels (O1, Oz, O2), with the exception of 0 vs. 135° and 90 vs. 135° contrasts at O2 channel, which were not significant. At parieto-occipital channels (PO7 and PO8) the differences did not reach significance, with the exception of 0° vs. 45° contrast. Summing up, orientation-related differences in Experiment 2 were significant only in the occipital area, but eventually we could replicate the findings of the previous experiment about the orientation-related N1b differences.

### Discussion for experiment 2

Results in this session reflect that reliable orientation vMMN with Gábor patches could be obtained with the largest possible (90°) orientation change. Oblique effect was found; vMMN to cardinal angles exhibited larger amplitudes and had 20 ms longer peak latencies. The vMMN to cardinal orientations had similar onset times than oblique vMMN, but its latency was prolonged due to larger amplitude and later offset. Orientation-related amplitude differences were present already around 120 ms, but the oblique effect could be observed only around 170 ms, in the time-interval of N1b component.

## General discussion

The main question of our study was whether an important feature of the visual system, the increased sensitivity for cardinal (horizontal and vertical) orientations (so-called oblique effect) influences pre-attentive processing reflected by the vMMN.

In our first experiment 50° deviancy did not elicit reliable vMMN. Nonetheless the largest possible orientation deviancy, 90° did elicit vMMN. The deviant-minus-standard difference wave was maximal over occipital areas between 120 and 200 ms, its peak falling between 134 and 162 ms. Stimuli changing from cardinal to cardinal orientations evoked longer and larger responses exhibiting a variant of the oblique effect.

Other studies investigating orientation vMMN obtained reliable vMMN in response to smaller deviances than we did. Czigler and Sulykos (2010) observed vMMN to bar stimuli changing from oblique to oblique orientations using 30 and 60° deviances. Astikainen et al. (2008) was able to register vMMN to 36° orientation changes for stimuli changing from oblique to oblique orientations. In the interference condition of the experiment of Sulykos et al. (2013) 30° deviances evoked vMMN. They were using similar Gábor stimuli as we did, but only in the lower visual field. Kimura et al. (2009, 2010a) presented 36° deviances and they also obtained vMMN. However, there is one important issue that we should consider. In our experiment every source of external orientation clues was removed. It was achieved by using a circular aperture over the screen, by providing no background light and by presenting stimuli in upper and lower visual field as well. In this way only orientation clues from the vestibular and somatosensory system remained available for the subjects. The studies mentioned above did not control this aspect, so it is possible that e.g., the outline of the computer screen facilitated the evaluation of orientation and the operation of automatic deviance detection.

It is a key question how we could interpret that we obtained increased response to cardinal changes. Although it was not directly assessed in lot of vMMN studies, presumably it is tenable assumption that the stronger the rule, the larger is the response to its violation, simply because change approximates the threshold of the vMMN system in more experimental trials. Representation of cardinal stimuli is more potent in the visual system, so their presence and deviations from them are more easily detected. While we were able to register electrophysiological responses reflecting fine differentiation of orientation between 150 and 190 ms over the occipital areas, and we could assume that the brain precisely mapped the orientation of Gábor patches, this occurred later than the vMMN, which appeared around 120 ms after stimulus onset. Still this argument does not account for why the response was also more sustained, and not just a simple amplitude differences was observed.

The oblique effect found in vMMN might correspond to the magnitude of deviance effect first observed in auditory processing (Näätänen et al., 1982; Sams et al., 1985, but see Horváth et al., 2008). In the case of the auditory MMN, larger deviancy between standard and deviant stimulus results in a MMN response with larger amplitudes and shorter latencies (Kujala et al., 2007). The existence of this phenomenon in visual domain is uncertain. Czigler and Sulykos (2010) obtained similar vMMNs to 30 and 60° deviancy with stimuli changing from oblique to oblique orientations. Maekawa et al. (2005) used windmill patterns, and according to their results, vMMN (or as they label it, “deviant-related negativity,” DRN) did not show increase of amplitude with increasing magnitude of deviance, only the latency decreased of the second negativity between 200 and 300 ms with maxima over temporal areas.

In our study, stimuli changing from cardinal to cardinal could be regarded as a stronger stimulus, and the perceived difference between them larger than the difference between oblique orientations, even though differences were the exact same in degrees. We interpret the sustained response to the more salient cardinal changes, as an indication of the visual system submitting more computational resources to changes that could be of larger importance.

Recently Cléry et al. (2013) found another version of magnitude of deviance effect using fMRI and a passive oddball paradigm. In their experiment the shape of the circular stimuli changed dynamically, for standard stimuli it stretched out horizontally into an ellipse, for deviant stimuli it stretched out vertically. The novel stimuli changed gradually to an irregular shape. The differences between responses elicited by deviant and novel stimuli were apparent in the visual cortex (BA 18 and 19) and in the medial frontal cortex (BA 8). In the anterior cingular cortex only novel stimuli evoked significant activity compared to baseline. Despite the fact that fMRI and ERP results are sometimes difficult to compare due to their widely different spatial and temporal resolution, in this case some parallels could be drawn. According to the authors extrastriatal differences might signal the activation of the visual areas that are responsible for the vMMN generation (or for other higher sensory processes), while differential fMRI response in the anterior cingular cortex might show the contribution of the areas responsible for the generation of the P3a component that is usually elicited by novel, non-target stimuli (Courchesne et al., 1975). However, because in this experiment the type of deviancy between the standard and deviant stimuli (vertical or horizontal stretching) was not the same as the deviancy between the deviant and novel stimuli (vertical stretching or changing to an irregular shape), it is difficult to compare their results with ours. Still, it seems that generators of the posterior part of vMMN are able to give not only all-or-nothing responses to visual deviancies.

We also found exogenous, orientation-related differences around 150–190 ms in the amplitude of the ERPs evoked by the standard stimuli. It is an important question why we observed these differences in a latency range which is quite late in time for visual orientation processing. The area V1 contains cells selective for orientation, and Gábor patches stimulate these as well. Visual processing in the striatal area (V1) is signaled by the C1 visual evoked potential, 50–90 ms after stimulus onset (Clark et al., 1995). Surprisingly, not too many studies reported (e.g., Song et al., 2010) orientation-related differences in this component. Unfortunately we were not able to examine this component due to simultaneous stimulation of upper and lower visual fields.

The first signs of orientation-related processing emerged between 100 and 140 ms in Experiment 2, where vertical (90°) stimuli elicited less negative N1a component than the other stimuli. Horizontal orientations evoked slightly different response than oblique stimuli. Arakawa et al. (2000) found oblique effect in the P100 component; at low spatial frequencies ERPs to cardinal orientations exhibited longer latencies than those to oblique stimuli, while at high spatial frequencies the relationship was reversed. Proverbio et al. (2002) reported orientation-related differences in P1 and P3 components, vertical elicited larger amplitudes than oblique stimuli (they did not look at horizontal stimuli). A study conducted by Yoshida et al. (1975) found differences in N1-P2 peak-to-peak amplitude, cardinal stimuli evoked larger responses than oblique stimuli. They obtained waveforms similar to ours using circular black and white gratings as stimuli, a P1-N1-P2 sequence was elicited with peak latencies of 110–120, 180–190, and 270–280 ms, respectively. Since they used only one active electrode (Oz), it is unclear whether N1 in their study had similar scalp topography as ours.

Our knowledge about the N1b wave is rather limited. This occipito-temporal component usually peaks approximately around 170–180 ms. In the experiment of Clark and Hillyard (1996) it was maximal at 180 ms, it was elicited by nontarget circular black and white checkerboards on contralateral sides. In our experiment this wave displayed bilateral distribution due to bilateral stimulus presentation. In the Clark and Hillyard (1996) study target stimuli evoked larger N1b responses, but the latency and scalp topography remained unaffected. The authors localized this component to the ventral-lateral visual cortex. This extrastriatal area is engaged in object identification and belongs to ventral visual pathway (Ungerleider and Haxby, 1994). This raises the possibility that in our experiments the visual system treated Gábor patches as objects and reprocessed the orientation of these objects during N1b. Others using everyday objects pointed out that processing of the orientation of objects could be tied to the dorsal occipito-parietal system (Valyear et al., 2006), so it is unclear what are the brain sources of the N1b component that we obtained in the present experiment. Examining the role of attention, Hopf et al. (2002) showed that a negativity with 165 ms peak amplitude is increased if subjects perform a discrimination task compared to simple detection. In our study Gábor patches were unattended, so presumably only detection took place, and the N1b modulation was clearly a result of difference in the physical characteristics of the stimuli.

To sum up the visual evoked potentials to standard stimuli provided evidence that orientation-related processing could be tracked until 190 ms after stimulus onset. Although vertical stimuli elicited different N1a in an earlier time interval (around 120 ms), N1b was the one that precisely mapped the orientation of stimuli. While theoretical assumptions suggest that Gábor patches are primarily processed in V1, it is possible that extrastriatal areas play a role in it as well—our findings corroborate this notion. The vMMN emerged earlier (onset time ~120 ms) than N1b (around 170 ms) in both cardinal and oblique stimuli conditions. It is possible that the precise orientation of the stimuli was achieved only after the process marked by N1b, and the visual deviance detection was not able to utilize this input. This could account for the widely different thresholds of visual deviance detection in the passive (90°) and active paradigm (10–17°).

The other feasible explanation is that the orientation of stimuli is determined in earlier levels of visual processing, possibly in V1, and the N1b component only signals the reprocessing of the stimulus as an object. In this case we can conclude that vMMN did not emerge to some of the differences that the visual system can detect, but only for considerably larger differences that exceed its own threshold.

Other studies provided further evidence that the sensitivity of active visual deviance detection is independent of the vMMN. In the experiment of Czigler et al. (2007) vMMN could be registered if the SOA between the stimulus and the backward mask was at least 40 ms. However if the stimulus—mask SOA was increased up to 174 ms, the magnitude of the vMMN remained the same. In attended conditions participants responded to deviants with a Go-NoGo response. In this case performance increased monotonically up to the longest stimulus-mask SOA (174 ms). Lyyra et al. (2012) combined change blindness paradigm and vMMN. Change blindness labels the phenomenon that human subjects are usually slow or unable to detect sudden, but minor changes in successive pictures of complex, natural scenes. The authors presented such pictures in oddball sequences while subjects tried to detect the change. They looked into the ERPs until the point when the detection of change happened. The authors hypothesized that vMMN will emerge even before the behavioral detection. Successful behavioral change detection occurred in the absence of vMMN using 500 ms inter-stimulus interval (ISI), probably because during this interval sensory memory crucial for vMMN elicitation decayed. With a shorter, 100 ms ISI, behavioral change detection was unchanged, but this time vMMN also emerged in posterior areas. What pertains to our question is that vMMN was not a necessary prerequisite of explicit change detection in their study. Our results also suggest similar dissociations; the processes responsible for the discrimination performance in the active paradigm are not the same as those generating vMMN.

While in the auditory modality, the threshold for MMN more or less corresponds to the behavioral threshold in an active, attended paradigm; in the visual domain it seems not to be the case. Alho et al. (1992) presented rectangular black and white gratings in their experiment, the deviant stimuli differed from standard in height. Only the larger of the two deviances elicited posterior negativity. In the study of Czigler et al. (2002) colored-black gratings were presented, and the results were similar: only larger deviancy evoked vMMN. In summary, this phenomenon was demonstrated with three different types of visual deviancy—shape, color, and orientation.

It is of particular interest what the functional significance of this dissimilar sensitivity is. The auditory MMN could serve as a basis of subsequent orienting response, and vMMN might have a similar role (see Czigler et al., 2006). It would not be functional if every discriminable change in a sequence elicited an orienting reaction, because it would lead to unnecessary distraction from the primary task. In addition, since humans gather information mainly from vision, the processing of stimuli in the focus of attention is substantial, and stimulation in the background is secondary. Auditory perception operates often outside the focus of attention, so automatic, unconscious perceptual processes might have a more central role than in vision.

### Conflict of interest statement

The authors declare that the research was conducted in the absence of any commercial or financial relationships that could be construed as a potential conflict of interest.

## Footnotes

^1^Although for the 0° vMMN the standards come from a block where stimuli changed from 0 to 50° (i.e., from cardinal to oblique), according to the widely accepted view in change detection studies, it is the deviancy that determines the condition, which in this case is the rare appearance of the 0° deviants in successions of 50° standards (i.e., a change from oblique to cardinal). This reasoning applies to every condition (50° condition, 90° condition, etc.)

^2^Accidentally we recorded ERPs to 167.5° instead of 157.5°, which would have been midmost between 135 and 180°.

^3^When e.g., standards were 22.5° it was possible that deviants had been 90° for one or more presentations, but as average thresholds (10° and 16.5°) indicates deviants were fluctuating around 10, 39, 100, and 129° for the 0, 22.5, 90, and 112.5° standard orientations, respectively. Each of these deviants represents oblique orientations.

^4^Our rationale for this change was to improve signal-to-noise ratio and achieve larger ERP responses. In Experiment1 we had considerably smaller evoked potentials, e.g., on Figure 3, at Oz channel ERPs are around 5.5 μV in amplitude, than in Experiment2, where the same ERPs were around 9 μV in amplitude (Figure 6).
